# Combined administration of fucoidan ameliorates tumor and chemotherapy-induced skeletal muscle atrophy in bladder cancer-bearing mice

**DOI:** 10.18632/oncotarget.9958

**Published:** 2016-06-13

**Authors:** Meng-Chuan Chen, Wen-Lin Hsu, Pa-An Hwang, Yen-Lin Chen, Tz-Chong Chou

**Affiliations:** ^1^ Graduate Institute of Medical Sciences, National Defense Medical Center, Taipei, Taiwan; ^2^ School of Medicine, Tzu Chi University, Hualien, Taiwan; ^3^ Department of Radiation Oncology, Buddhist Tzu Chi General Hospital, Hualien, Taiwan; ^4^ Department of Bioscience and Biotechnology, National Taiwan Ocean University, Keelung, Taiwan; ^5^ Department of Pathology, Cardinal Tien Hospital, School of Medicine, Fu-Jen Catholic University, New Taipei City, Taiwan; ^6^ Institute of Medical Sciences, Tzu Chi University, Hualien, Taiwan; ^7^ Department of Biotechnology, Asia University, Taichung, Taiwan; ^8^ China Medical University Hospital, China Medical University, Taichung, Taiwan

**Keywords:** fucoidan, cancer cachexia, muscle atrophy, chemotherapy, inflammation

## Abstract

Cancer cachexia is characterized by anorexia, skeletal muscle atrophy, and systemic inflammation. Fucoidan extracted from brown algae exhibits anti-inflammatory and anticancer activities. However, whether fucoidan ameliorates tumour and chemotherapy-induced muscle atrophy and -related cachectic symptoms remains unknown. Compared with mice with bladder cancer treated with chemotherapy alone (TGC group), those treated with a combination of low molecular weight fucoidan (LMWF) and chemotherapy drugs such as gemcitabine and cisplatin (TGCF) showed a significant reduction of body weight loss, muscle atrophy, and intestinal injury and dysfunction. Moreover, myostatin, activin A, and pro-inflammatory cytokine production, FoxO3 expression and activation, NF-κB activation, MuRF-1 and MAFbx/atrogin-1 expression, and proteasome activity in muscle were significantly decreased in the TGCF group compared with the TGC group. In addition, insulin-like growth factor 1 (IGF-1) expression and formation, and IGF-1-regulated mTOR/p70S6k/4EBP-1 protein synthesis signalling were elevated in the TGCF group compared with the TGC group. Taken together, these results suggest that LMWF is a potential agent for preventing cancer cachexia-associated muscle atrophy during chemotherapy. Furthermore, the beneficial effect of LMWF may be attributed to suppressing NF-κB-evoked inflammation, myostatin and activin A production, and subsequent muscle proteolysis, and enhancing IGF-1-dependent protein synthesis.

## INTRODUCTION

More than 80% of patients with advanced cancer suffer from cachexia, which contributes to at least 20% of deaths in cancer patients [1, 2]. Cancer cachexia is characterized by severe body weight loss, skeletal muscle atrophy, anorexia, fatigue, inflammation, and abnormal metabolism, resulting in a significant decrease in the quality of life, and poor tolerance to conventional cancer treatments such as chemotherapy [3]. Steadily progressive loss of skeletal muscle mass is the most prominent feature of cachexia. Despite the use of the currently available nutritional supplements or medications, muscle atrophy remains a major problem in clinical therapy [4, 5]. Muscle proteolysis is predominately mediated by the ubiquitin (Ub) proteasome system (UPS) [6], and atrophying muscles show elevated UPS activity [7]. Activation of the forkhead box O (FoxO), a transcription factor, can increase proteasome activity through up-regulation of muscle-specific Ub-conjugating E3 ligases such as F-box (MAFbx)/atrogin-1 and muscle ring finger 1 (MuRF-1). Conversely, MAFbx or MuRF-1 deficient mice exhibit a marked resistance to muscle atrophy [6]; therefore, targeting UPS can be an effective strategy for ameliorating muscle wasting. Furthermore, myostatin and activin overproduction, nuclear factor-κB (NF-κB)-evoked inflammation, impaired insulin-like growth factor-1 (IGF-1)-induced protein synthesis in the host, and the interactions between the tumor and host systems all contribute to the development of cancer cachexia-associated muscle atrophy [8, 9].

Accordingly to an epidemiological study, 23% of patients with bladder cancer had clear signs of cancer-related body weight loss [10]. Consistently, skeletal muscle atrophy is often observed in animal bladder cancer [11]. Currently, a combination treatment with gemcitabine (2′, 2′-difluorodeoxycytidine, Gemzar) and cisplatin (cis-diammine-dichloroplatinum) is considered the standard regimen for advanced, metastatic bladder cancer therapy [12, 13]. However, patients treated with chemotherapy drugs, particularly cisplatin, frequently experience adverse effects such as nephrotoxicity, ototoxicity, gastrointestinal injury, and body weight loss, which limiting the clinical application of these drugs [14, 15].Therefore, developing more safe and effective drugs or supplements for preventing chemotherapy-induced cytotoxicity and side effects is necessary.

**Figure 1:**
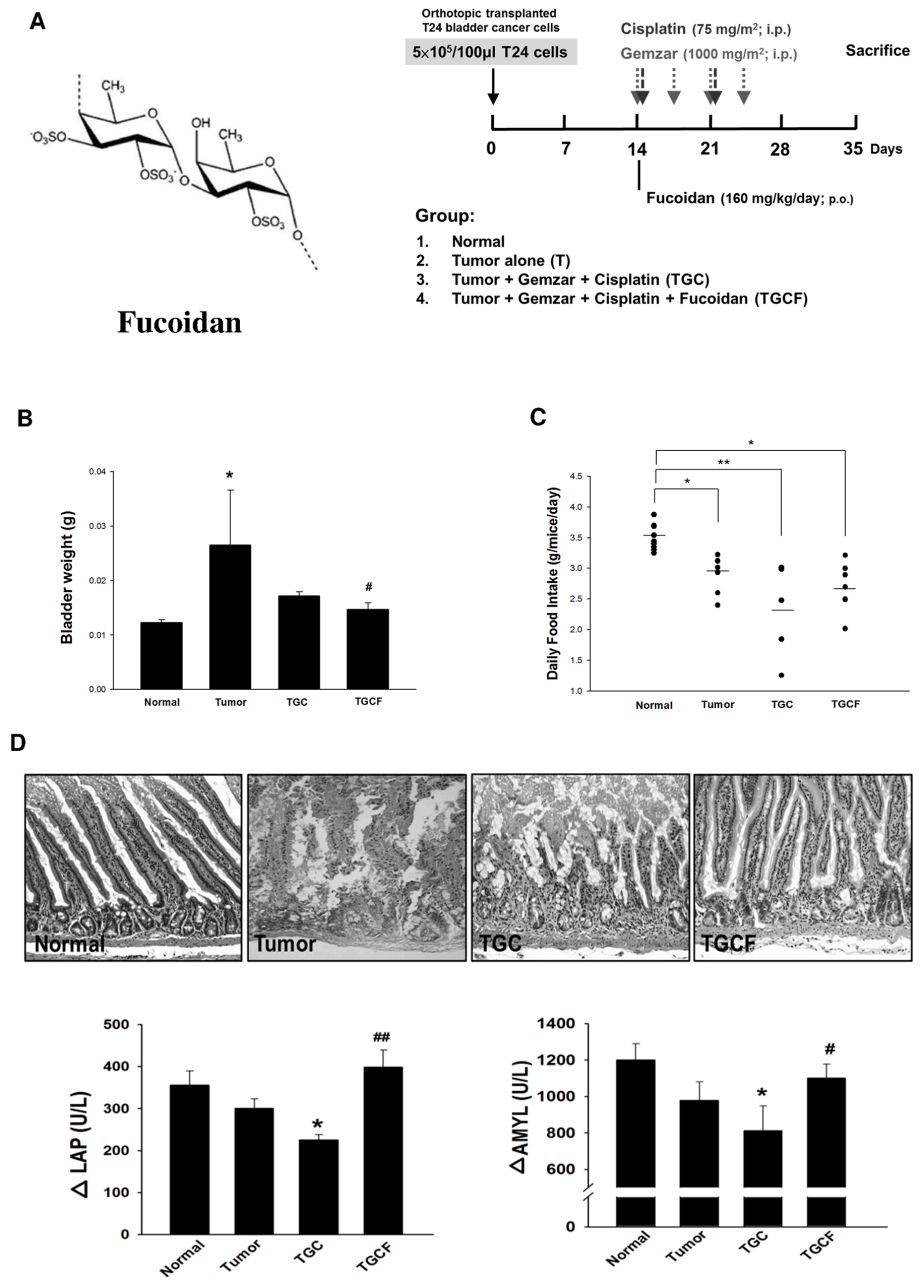
Effects of LMWF on tumor growth, food intake and intestinal damage and function The LMWF structure and experimental design was shown **A.** The bladder weight **B.** daily food intake **C.** the morphological changes and the digestive enzyme activity of intestines **D.** in different groups were determined. Data was expressed as mean ± SEM (n=5-10). *P < 0.05, **P < 0.01 versus normal group; #*P* < 0.05, ##*P* < 0.01 versus TGC group.

Fucoidan (Figure 1A), a class of fucose-enriched sulphated polysaccharides, is isolated from brown algae. Fucoidan has recently drawn considerable attention because it has several beneficial functions, including anti-inflammatory and anticancer activities [16–18]. Furthermore, the functions of fucoidan are mainly dependent on its molecular weight, sulphate content, and the algae species it is isolated from. Generally, low molecular weight fucoidan (LMWF) has a greater anticancer activity [19]. To date, the effects of LMWF on cancer cachectic symptoms have not yet been elucidated. Therefore, the present study investigated whether LMWF supplementation ameliorates chemotherapy-induced muscle atrophy in mice with bladder cancer, and elucidated the underlying molecular mechanisms.

## RESULTS

### LMWF reduces tumour growth, and intestinal injury and dysfunction

After orthotopic implantation of T24 cells into the bladder for two weeks, mice were treated with different combinations of drugs for 21 days, and subsequent tests were performed according to the study design (Figure 1A). The mice were divided into following four groups: the (1) normal group; (2) T group (tumour alone group); (3) TGC group: a standard diet and an intraperitoneal injection of gemcitabine (1000 mg/m^2^ per 3 days) and cisplatin (75 mg/m^2^/week); and (4) TGCF group: a standard diet plus LMWF (160 mg/kg/day, p.o.), and an intraperitoneal injection of gemcitabine and cisplatin. The TGCF group exhibited a stronger anticancer effect than that of the TGC group, as evidenced by the reduction of bladder weight, which reflects the tumour growth (Figure 1B). The TGC group had the least food intake, and the food intake was increased by LMWF treatment (Figure 1C). In addition, compared with the TGC group, intestinal mucosal structure damage and impaired digestive enzyme activity such as leucine amino peptidase (LAP), a digestion enzyme for peptides, and amylase (AMYL), a digestion enzyme for sugars, were greatly improved in the TGCF group (Figure 1D).

### LMWF attenuates mortality, muscle atrophy, and proteasome activity

**Figure 2:**
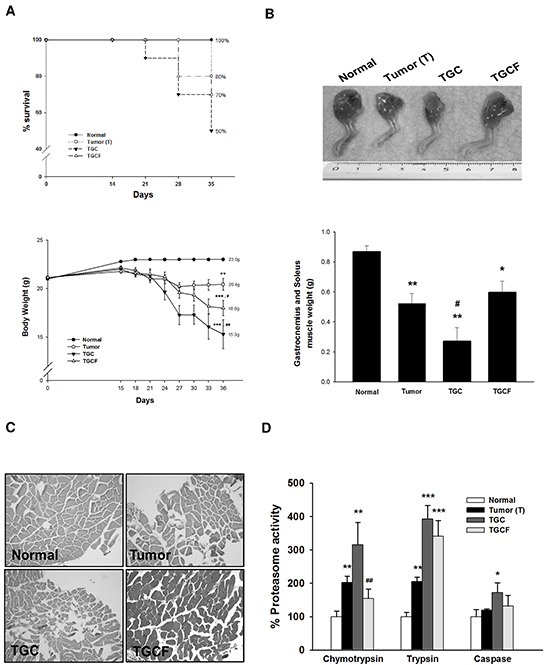
Effects of LMWF on survival rate and muscle atrophy The survival rate and body weight **A.** a representative image of the muscle of limb, and the weight of gastrocnemius and soleus muscle were photographed or measured **B.** The morphological changes of gastrocnemius muscle stained with H&E (magnification, ×200) **C.** and the proteasome activity **D.** in muscle were determined. Data was expressed as mean ± SEM (n=5-10). *P < 0.05, **P < 0.01, ***P < 0.001 versus normal group. ##*P* < 0.01 versus TGC group.

The survival rate was higher in the TGCF group than in the TGC group (70% vs 50%) (Figure 2A). At the end of the study, the mice in the T group lost 5.2±0.9% of their initial body weight, whereas those in the normal group gained body weight. The mice in the TGC and TGCF groups lost 28.3±1.8%, and 16.2±1.2% of their initial body weight, respectively (Figure 2A). Similarly, compared with the TGC group, the loss of gastrocnemius and soleus muscle mass (Figure 2B), and muscle atrophy evaluated by histological examination (Figure 2C) were remarkably reduced in the mice of TGCF group. Moreover, a marked increase of muscular proteasome activity, particularly chymotrypsin, was observed in the TGC group mice, whereas this activity was attenuated in the TGCF group mice (Figure 2D).

### LMWF down-regulates muscle wasting-related genes

**Figure 3:**
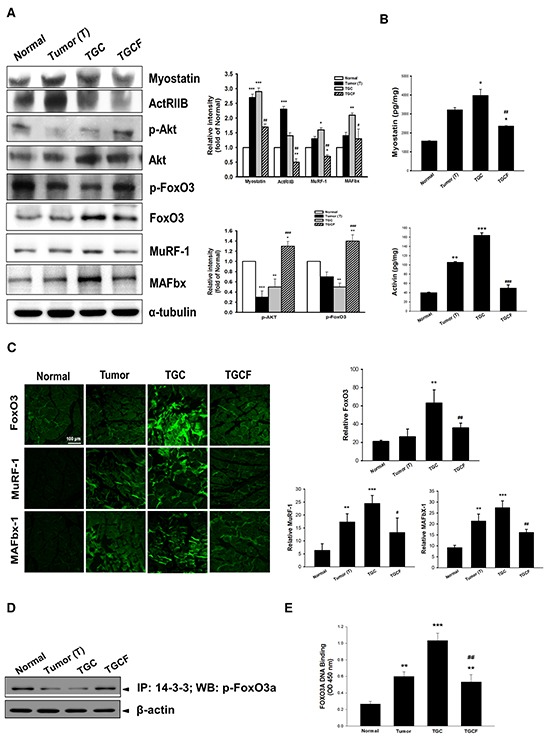
Effects of LMWF on muscle proteolysis-related gene expression The atrogenic gene expression **A.** and the formation of myostatin, and activin A **B.** in muscle were determined. The amounts of FoxO3, MuRF-1 and MAFbx determined by immunofluorescence staining assay **C.** and the association of p-FoxO3a with 14-3-3 chaperone protein **D.** FoxO3a transcription factor activity **E.** in skeletal muscle were examined. Data was expressed as mean ± SEM (n=5-10). *P < 0.05, **P < 0.01, ***P < 0.001 versus normal group. #*P* < 0.05, ##*P* < 0.01, ###*P* < 0.001 versus TGC group.

The up-regulation of ActRIIB, FoxO3, MuRF 1, and MAFbx, as well as the down-regulation of p-Akt and p-FoxO3 accompanied by myostatin and activin A overproduction in the muscle of the T and TGC group mice were significantly reversed by the combination treatment with LMWF (Figure 3A and 3B). Similarly, immunofluorescence staining revealed that FoxO3, MuRF 1, and MAFbx expression was markedly elevated, particularly in the muscle of the TGC group mice, but it was greatly suppressed in the mice of TGCF group (Figure 3C). Additionally, the interaction of FoxO3 and the 14-3-3 chaperone protein, examined through an immunoprecipitation assay, was remarkably higher in the TGCF group than in the T and TGC groups (Figure 3D). As expected, a significant increase of FoxO3 activity, examined by using a FOXO3 DNA binding kit, was observed in the TGC group, whereas it was markedly reduced in the TGCF group (Figure 3E).

### LMWF inhibits inflammatory molecule formation and NF-κB activation

**Figure 4:**
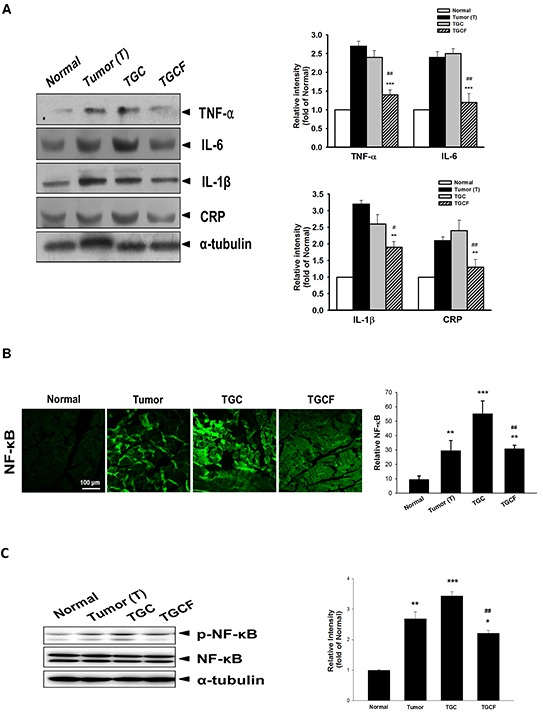
Effects of LMWF on pro-inflammatory cytokine expression and NF-κB activation The protein levels of pro-inflammatory cytokines, CRP, **A.** and NF-κB **B.** and phospho-NF-κB **C.** in muscle were determined by immunofluorescence staining or Western blotting assay. Data was expressed as mean ± SEM (n=5-10). *P < 0.05, **P < 0.01, ***P < 0.001 versus normal group. #*P* < 0.05, ##*P* < 0.01 versus TGC group.

Systemic inflammation is a critical factor causing the development of cancer cachexia [20]. Compared with the T and TGC group mice, the expression of pro-inflammatory cytokines, including TNF-α, IL-6, IL-1β, and C-reactive protein (CRP) (Figure 4A), and NF-κB and p-NF-κB in the muscle determined by immunofluorescence staining (Figure 4B) or Western blotting (Figure 4C) assays were diminished after LMWF treatment.

### LMWF activates IGF-1-regulated signalling

**Figure 5:**
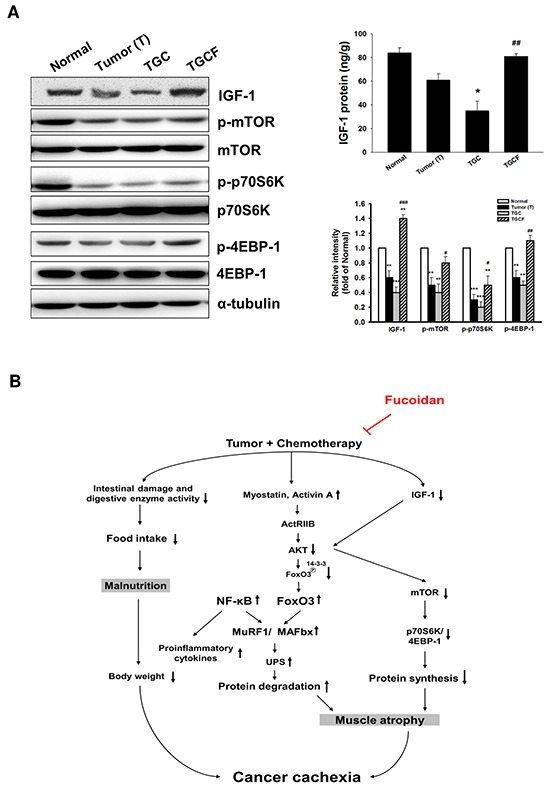
Effects of LMWF on IGF-1-regulated signalling and the proposed mechanisms accounting for the anti-cachectic activity The IGF-1-regulated protein synthesis-related signalling pathway and IGF-1 generation in muscle of various groups were determined **A.** Data was expressed as mean ± SEM (n=5-10). *P < 0.05, **P < 0.01, ***P < 0.001 versus normal group. #*P* < 0.05, ##*P* < 0.01, ###*P* < 0.001 versus TGC group. Combined treatment with LMWF inhibits myostatin/activin A/FoxO3 signaling, NF-κB activation, and pro-inflammatory cytokine formation, which in turn attenuates MAFbx and MuRF1 expression and UPS-induced protein degradation. Meanwhile, LMWF also can elevate IGF-1 formation and its regulated protein synthesis, and prevent intestinal damage and dysfunction. These actions of LMWF ultimately ameliorate tumor and chemotherapy-induced skeletal muscle atrophy **B.**

As shown in Figure 5A, IGF-1 expression and production, and IGF-1-regulated p-mammalian target of rapamycin (p-mTOR)/p-p70S6K/p-4EBP-1 signalling were significantly increased in the TGCF group compared with the TGC group.

## DISCUSSION

This is the first study to demonstrate that LMWF supplementation alleviates chemotherapy-induced cachectic symptoms, particularly skeletal muscle atrophy in mice with bladder cancer. Several tumour and host factors can induce muscle atrophy, mainly by increasing muscle proteolysis, or reducing protein synthesis, or both [9]. Myostatin, belonging to the transforming growth factor-β (TGF-β) superfamily, is predominately expressed in the skeletal muscle. It can inhibit skeletal muscle growth by suppressing myoblast proliferation and myogenesis [21]. In addition, activin A has been regarded as another crucial stimulator for muscle atrophy. Overproduction of myostatin and activin A is often seen in cancer patients suffering from cachexia and experimental cancer cachexia models [22, 23]. Conversely, blocking myostatin with neutralizing antibodies or antagonists greatly increases the muscle size and physical strength [24]. Upon activation, both myostatin and activins bind to the same muscle surface receptor complex that is composed of type-II activin receptors (ActRIIA and ActRIIB) and type-I activin receptors (ALK4 and ALK5), thereby triggering muscle protein degradation through the UPS [24]. The ubiquitination of protein substrate is controlled by a series of enzymes. First, Ub is activated by Ub-activating enzyme (E1), and then transferred to the active site of Ub carrier protein by Ub-conjugating enzyme (E2). Subsequently, Ub-protein ligases (E3) catalyse a conjugation reaction that results in polyubiquitination of the protein substrate. Only the Ub-targeted protein can be recognized by proteasome, and processed into smaller peptides. MAFbx/atrogin-1 and MuRF-1 are key E3 Ub-ligases accounting for the muscle protein degradation under cachexia conditions [25], and FoxO3 is primarily responsible for the induction of MAFbx/atrogin-1 and MuRF-1. Notably, FoxO activity is modulated by the change in subcellular localization and degradation of the FoxO protein. Under normal condition, FoxO is phosphorylated by Akt, and the phosphorylated FoxO is exported from the nucleus to the cytoplasm and degraded by proteasome in a chaperone 14-3-3-dependent process [26]. Myostatn and activin A activation reduces Akt activity, which leads to an accumulation of dephospho-FoxO, the active form of FoxO, thereby activating MuRF-1 and MAFbx transcription. Our results confirmed that combination treatment with LMWF significantly suppressed the myostatin/activin A/FoxO3/MuRF-1/MAFbx signalling, and proteasome activity in the muscle of the TGC group mice, which may be due to up-regulation of p-Akt and p-FoxO3. As expected, administration of LMWF strongly increased the association of 14-3-3 with phospho-FoxO3 and inhibited FoxO3 transcriptional activity. Moreover, maintaining intestinal structure and function is essential for nutritional intake and body growth. After LMWF treatment, the intestinal damage and dysfunction and decreased food intake observed in the TGC group mice were alleviated. Therefore, LMWF-mediated reduction of muscle atrophy and body weight loss may be, at least in part, attributed to the attenuation of the UPS and intestinal dysfunction.

Previous studies have demonstrated that systemic inflammation, particularly NF-κB-evoked pro-inflammatory cytokine formation, plays a critical role in inducing muscle wasting by activating the UPS through inhibition of Akt activity and impairment of muscle differentiation and myogenesis [27–29]. The levels of pro-inflammatory cytokines in the serum and NF-κB activation are dramatically increased in patients with cancer cachexia [28]. Based on our findings that LMWF treatment greatly reduced NF-κB activation, pro-inflammatory cytokine formation, and CRP expression, a biomarker of systemic inflammation [30], the anti-inflammatory activity of LMWF may also contribute to its anti-cachectic effect.

Finally, we investigated whether LMWF activates protein synthesis-related signalling pathway. Several studies have indicated that IGF-1 can stimulate protein synthesis and muscle growth by activating PI3K/Akt/mTOR/p70S6K/4EBP-1 signalling [31–33]. In addition, IGF-1 has an inhibitory effect on proteolysis through the suppression of FoxO accumulation and activation [32]. Therefore, IGF-1 not only reduces muscle protein degradation but also increases protein synthesis. A novel finding of this study is that decreased IGF-1 expression and production and down-regulation of mTOR/p70S6K/4EBP-1 signalling observed in the TGC group mice were markedly reversed in the TGCF group. Previous studies have proposed that elevation of myostatin and pro-inflammatory cytokines may inhibit IGF-1 bioavailability and IGF-1-regulated signalling [34, 35]. Accordingly, LMWF-induced IGF-1 expression and IGF-1-dependent signalling are possibly associated with the inhibition of myostatin formation and inflammatory responses. In summary, we demonstrated that administration of LMWF ameliorated tumour and chemotherapy-induced muscle atrophy and -related cachectic symptoms. The molecular mechanisms through which LMWF prevents muscle mass loss may be largely attributed to the reduction of UPS-induced muscle proteolysis by suppressing the myostatin/activin A /FoxO3/MAFbx/MuRF-1 cascade and NF-κB-evoked inflammation, and activating IGF-1-induced protein generation, as well as preventing intestinal damage and dysfunction (Figure 5B). Therefore, we can conclude that LMWF may be a promising nutritional supplement or chemotherapeutic adjuvant for minimizing chemotherapy-induced muscle atrophy and toxicity.

## MATERIALS AND METHODS

### LMWF preparation and reagents

The LMWF was a gift from Hi-Q Marine Biotech International Ltd (Taiwan) and prepared as described previously [36]. Briefly, the fresh dried *Sargassum hemiphyllum* was boiled for 30 min. Then, the hot water extract was lyophilized and 4 volumes of 95% ethanol were added for overnight at 4°C. After the sample was incubated with glycolytic enzyme for 6 h, the supernatants were passed through a series of molecular weight cut-off membranes to obtainthe LMWF that was mainly 760 Da. The antibodies including anti-TNF-α, anti-IL-1β, anti-IGF-1, anti-MuRF-1, anti-MAFbX-1 and anti-β-actin were purchased from Santa Cruz Biotechnology (CA, USA). The anti-AKT, anti-phospho-AKT, anti-phospho-NF-κB, anti-NF-κB, anti-phospho-FoxO3, anti-FoxO3, anti-mTOR, anti-phospho-mTOR, antip70S6K, anti-phospho-p70S6K, anti-4E-BP-1 and anti-phospho-4E-BP-1 were purchased from Cell Signaling Technology (Danvers, MA, USA). The anti-CRP was purchased from NOVUS Biologicals (CO, USA), and the anti-myostatin, anti-IL-6, anti-TNF-α, anti-IL-1β were purchased from GeneTex, Inc. (CA, USA). Horseradish peroxidase (HRP)-labeled secondary antibody was obtained from Abcam (Cambridge, MA, USA). Cisplatin and gemcitabine were provided by Eli Lilly (Indianapolis, IN, USA). The enzyme-linked immunosorbent assay (ELISA) kits of myostatin, activin A, and IGF-1 were purchased from R&D Systems, Inc. (MN, USA). Other reagents were purchased from Sigma-Aldrich Corporation (St. Louis, MO, USA).

### Cell culture

The T24 human bladder cancer cell was purchased from the Bioresource Collection and Research Center (Taipei, Taiwan). T24 cells were incubated in RPMI1640 (Thermo Fisher Scientific Inc, Waltham, Utah, USA) supplemented with 10 % fetal bovine serum (Thermo Fisher Scientific Inc), 2 mmol/L L-glutamine, and 100 U/mL penicillin-streptomycin (Gibco, Carlsbad, St. CA, USA). Cells were maintained in an incubator with room air: CO_2_ (95:5, v/v) at 37°C.

### Animal model

The 7-week-old female athymic nude mice (BALB/c) weighing ~25 g were used for the study. The animal care and experimental procedures were conducted in accordance with the Guiding Principle in the Care and Use of Animals and approved by the Institutional Animal Care and Use Committee of National Defense Medical Center (IACUC 12156). The mice were anaesthetized with an intraperitoneal injection of ketamine HCl/xylazine (100 mg/15 mg body weight per mouse) for subsequent procedure. The bladder was catheterized via the urethra with a 24 gauge plastic intravenous cannula under sterile conditions. Then, the bladder was instilled with 0.1 ml 0.1 N HCl solution for 15 seconds, and neutralized with 0.1 ml 0.1 N KOH. After the HCl and KOH were squeezed out from the bladder, sterile saline was flushed, followed by an instillation of 100 μl of T24 cells (5×10^5^) via the cannula. In this study, there were four weight-matched groups as described in Results. Originally, the number of mice of each group was 10. Because the high mortality of cancer cachexia, at the end of the study, the survival rate of tumor alone or tumor-treated groups was 50% - 80%. Thus, the final number of animals used for subsequent tests of different groups was 5-10 mice.

### Histological examination and immunohistochemical staining

Tissues were fixed with 10% formaldehyde and embedded in paraffin followed by H&E staining to evaluate the pathological changes. For immunohistochemical assay, muscle tissue sections were incubated with various primary antibody of target genes for overnight followed by addition of fluorescein isothiocyanate (FITC)-coupled secondary antibody (1:200, Abcam Cambridge, MA, USA) for 1 h. After extensive washings with PBST, the coverslips were mounted onto the glass slides and photographed with a fluorescence microscope (Leica, Welzar, Germany). The intensity of immunoreactivity was measured by using densitometer and MetaMorph image analysis software.

### Intestinal digestion enzyme activity assay

The intestinal extracts from jejunum were prepared in 0.9% NaCl supplemented with proteinase inhibitors. Then, the samples were used to measure the major intestinal digestion enzyme activity such as LAP and AMYL according to the manufacturer's instruction (Fuji Photo Film Co. Ltd., Tokyo, Japan) [37].

### Proteasome activity assay

The skeletal muscle (gastrocnemius muscle) samples were rinsed in ice-cold phosphate-buffered saline (PBS) to remove blood. The proteasome activity in muscle including chymotrypsin, trypsin and caspase was determined by using commercially available Proteasome-Glo™ 3-Substrate Systems kit [38].

### Western blotting

The protein samples (100 μg) were separated on a 10% SDS-PAGE, and transferred onto nitrocellulose membranes. After blocking with 5% nonfat dry milk in 5% TBST for 1 h, the membranes were incubated with various appropriately diluted primary antibody for target genes at 4°C for overnight. After washing with TBST, the membranes were incubated with horseradish peroxidase-conjugated secondary antibody for 1 h and the immunoreactivity was visualized using enhanced HRP substrate luminol reagent (Milipore, Billerica, MA, USA).

### FOXO3A DNA binding assay

The transcription factor activity of FoxO3 was determined using FoxO3 DNA binding ELISA kit (LifeSpan BioSciences, Seattle, WA, USA) according to the manufacturer's instructions.

### Statistical analysis

The experimental data were expressed as the mean±SEM. One-way ANOVA with *post hoc* Bonferroni test was used for statistical analysis. Results were considered significant difference at a value of *P* < 0.05.

## References

[1] Tuca A, Jimenez-Fonseca P, Gascon P (2013). Clinical evaluation and optimal management of cancer cachexia. Crit Rev Oncol Hematol.

[2] Coss CC, Bohl CE, Dalton JT (2011). Cancer cachexia therapy: a key weapon in the fight against cancer. Curr Opin Clin Nutr Metab Care.

[3] Fearon K, Strasser F, Anker SD, Bosaeus I, Bruera E, Fainsinger RL, Jatoi A, Loprinzi C, MacDonald N, Mantovani G, Davis M, Muscaritoli M, Ottery F (2011). Definition and classification of cancer cachexia: an international consensus. Lancet Oncol.

[4] Douglas E, McMillan DC (2014). Towards a simple objective framework for the investigation and treatment of cancer cachexia: the Glasgow Prognostic Score. Cancer Treat Rev.

[5] Donohoe CL, Ryan AM, Reynolds JV (2011). Cancer cachexia: mechanisms and clinical implications. Gastroenterol Res Pract.

[6] de Palma L, Marinelli M, Pavan M, Orazi A (2008). Ubiquitin ligases MuRF1 and MAFbx in human skeletal muscle atrophy. Joint Bone Spine.

[7] Sandri M (2008). Signaling in muscle atrophy and hypertrophy. Physiology (Bethesda).

[8] Wing SS, Lecker SH, Jagoe RT (2011). Proteolysis in illness-associated skeletal muscle atrophy: from pathways to networks. Crit Rev Clin Lab Sci.

[9] Fearon KC, Glass DJ, Guttridge DC (2012). Cancer cachexia: mediators signaling and metabolic pathways. Cell Metab.

[10] Dempsey DT, Buzby GP, Mullen JL (1983). Nutritional assessment in the seriously ill patient. J Am Coll Nutr.

[11] Padrao AI, Oliveira P, Vitorino R, Colaco B, Pires MJ, Marquez M, Castellanos E, Neuparth MJ, Teixeira C, Costa C, Moreira-Goncalves D, Cabral S, Duarte JA (2013). Bladder cancer-induced skeletal muscle wasting: disclosing the role of mitochondria plasticity. Int J Biochem Cell Biol.

[12] Cohen MH, Rothmann M (2001). Gemcitabine and cisplatin for advanced metastatic bladder cancer. J Clin Oncol.

[13] von der Maase H, Sengelov L, Roberts JT, Ricci S, Dogliotti L, Oliver T, Moore MJ, Zimmermann A, Arning M (2005). Long-term survival results of a randomized trial comparing gemcitabine plus cisplatin with methotrexate vinblastine doxorubicin plus cisplatin in patients with bladder cancer. J Clin Oncol.

[14] Fanzani A, Zanola A, Rovetta F, Rossi S, Aleo MF (2011). Cisplatin triggers atrophy of skeletal C2C12 myotubes via impairment of Akt signalling pathway and subsequent increment activity of proteasome and autophagy systems. Toxicol Appl Pharmacol.

[15] Karasawa T, Steyger PS (2015). An integrated view of cisplatin-induced nephrotoxicity and ototoxicity. Toxicol Lett.

[16] Cumashi A, Ushakova NA, Preobrazhenskaya ME, D'Incecco A, Piccoli A, Totani L, Tinari N, Morozevich GE, Berman AE, Bilan MI, Usov AI, Ustyuzhanina NE, Grachev AA (2007). A comparative study of the anti-inflammatory anticoagulant antiangiogenic and antiadhesive activities of nine different fucoidans from brown seaweeds. Glycobiology.

[17] Zorofchian Moghadamtousi S, Karimian H, Khanabdali R, Razavi M, Firoozinia M, Zandi K, Abdul Kadir H (2014). Anticancer and antitumor potential of fucoidan and fucoxanthin two main metabolites isolated from brown algae. ScientificWorldJournal.

[18] Senthilkumar K, Manivasagan P, Venkatesan J, Kim SK (2014). Brown seaweed fucoidan: biological activity and apoptosis growth signaling mechanism in cancer. Int J Biol Macromol.

[19] Cumashi A, Ushakova NA, Preobrazhenskaya ME, D'Incecco A, Piccoli A, Totani L, Tinari N, Morozevich GE, Berman AE, Bilan MI, Usov AI, Ustyuzhanina NE, Grachev AA (2007). A comparative study of the anti-inflammatory anticoagulant antiangiogenic and antiadhesive activities of nine different fucoidans from brown seaweeds. Glycobiology.

[20] Aoyagi T, Terracina KP, Raza A, Matsubara H, Takabe K (2015). Cancer cachexia mechanism and treatment. World J Gastrointest Oncol.

[21] Han HQ, Mitch WE (2011). Targeting the myostatin signaling pathway to treat muscle wasting diseases. Curr Opin Support Palliat Care.

[22] Aversa Z, Bonetto A, Penna F, Costelli P, Di Rienzo G, Lacitignola A, Baccino FM, Ziparo V, Mercantini P, Rossi Fanelli F, Muscaritoli M (2012). Changes in myostatin signaling in non-weight-losing cancer patients. Ann Surg Oncol.

[23] Costelli P, Muscaritoli M, Bonetto A, Penna F, Reffo P, Bossola M, Bonelli G, Doglietto GB, Baccino FM, Rossi Fanelli F (2008). Muscle myostatin signalling is enhanced in experimental cancer cachexia. Eur J Clin Invest.

[24] Elkina Y, von Haehling S, Anker SD, Springer J (2011). The role of myostatin in muscle wasting: an overview. J Cachexia Sarcopenia Muscle.

[25] Yuan L, Han J, Meng Q, Xi Q, Zhuang Q, Jiang Y, Han Y, Zhang B, Fang J, Wu G (2015). Muscle-specific E3 ubiquitin ligases are involved in muscle atrophy of cancer cachexia: an in vitro and in vivo study. Oncol Rep.

[26] Tzivion G, Dobson M, Ramakrishnan G (2011). FoxO transcription factors; Regulation by AKT and 14-3-3 proteins. Biochim Biophys Acta.

[27] Op den Kamp CM, Langen RC, Snepvangers FJ, de Theije CC, Schellekens JM, Laugs F, Dingemans AM, Schols AM (2013). Nuclear transcription factor kappa B activation and protein turnover adaptations in skeletal muscle of patients with progressive stages of lung cancer cachexia. Am J Clin Nutr.

[28] Deans C, Wigmore SJ (2005). Systemic inflammation cachexia and prognosis in patients with cancer. Curr Opin Clin Nutr Metab Care.

[29] Onesti JK, Guttridge DC (2014). Inflammation based regulation of cancer cachexia. Biomed Res Int.

[30] Zheng Z, Zhou L, Gao S, Yang Z, Yao J, Zheng S (2013). Prognostic role of C-reactive protein in hepatocellular carcinoma: a systematic review and meta-analysis. Int J Med Sci.

[31] Velloso CP (2008). Regulation of muscle mass by growth hormone and IGF-I. Br J Pharmacol.

[32] Stitt TN, Drujan D, Clarke BA, Panaro F, Timofeyva Y, Kline WO, Gonzalez M, Yancopoulos GD, Glass DJ (2004). The IGF-1/PI3K/Akt pathway prevents expression of muscle atrophy-induced ubiquitin ligases by inhibiting FOXO transcription factors. Mol Cell.

[33] Ge X, Zhang Y, Jiang H (2013). Signaling pathways mediating the effects of insulin-like growth factor-I in bovine muscle satellite cells. Mol Cell Endocrinol.

[34] Zhou X, Wang JL, Lu J, Song Y, Kwak KS, Jiao Q, Rosenfeld R, Chen Q, Boone T, Simonet WS, Lacey DL, Goldberg AL, Han HQ (2010). Reversal of cancer cachexia and muscle wasting by ActRIIB antagonism leads to prolonged survival. Cell.

[35] Lazarus DD, Moldawer LL, Lowry SF (1993). Insulin-like growth factor-1 activity is inhibited by interleukin-1 alpha tumor necrosis factor-alpha and interleukin-6. Lymphokine Cytokine Res.

[36] Chen MC, Hsu WL, Hwang PA, Chou TC (2015). Low Molecular Weight Fucoidan Inhibits Tumor Angiogenesis through Downregulation of HIF-1/VEGF Signaling under Hypoxia. Mar Drugs.

[37] Arivarasu NA, Fatima S, Mahmood R (2007). Effect of cisplatin on brush border membrane enzymes and anti-oxidant system of rat intestine. Life Sci.

[38] Strucksberg KH, Tangavelou K, Schroder R, Clemen CS (2010). Proteasomal activity in skeletal muscle: a matter of assay design muscle type and age. Anal Biochem.

